# Supplementary material for: Yiai Fuzheng decoction inhibits triple-negative breast cancer by remodeling the immune microenvironment
Source: Front Immunol. 2025 Sep 30;16:1615631. doi: 10.3389/fimmu.2025.1615631 (PMC12518410; doi:10.3389/fimmu.2025.1615631)
Supplement: Supplementary file 2 [file DataSheet1.zip › Supplementary File 2/Treatment/C_vs_D_vs_H/06_Pathway_Analysis/Enrichment_Analysis/smpdb_pathway/Network.pdf]

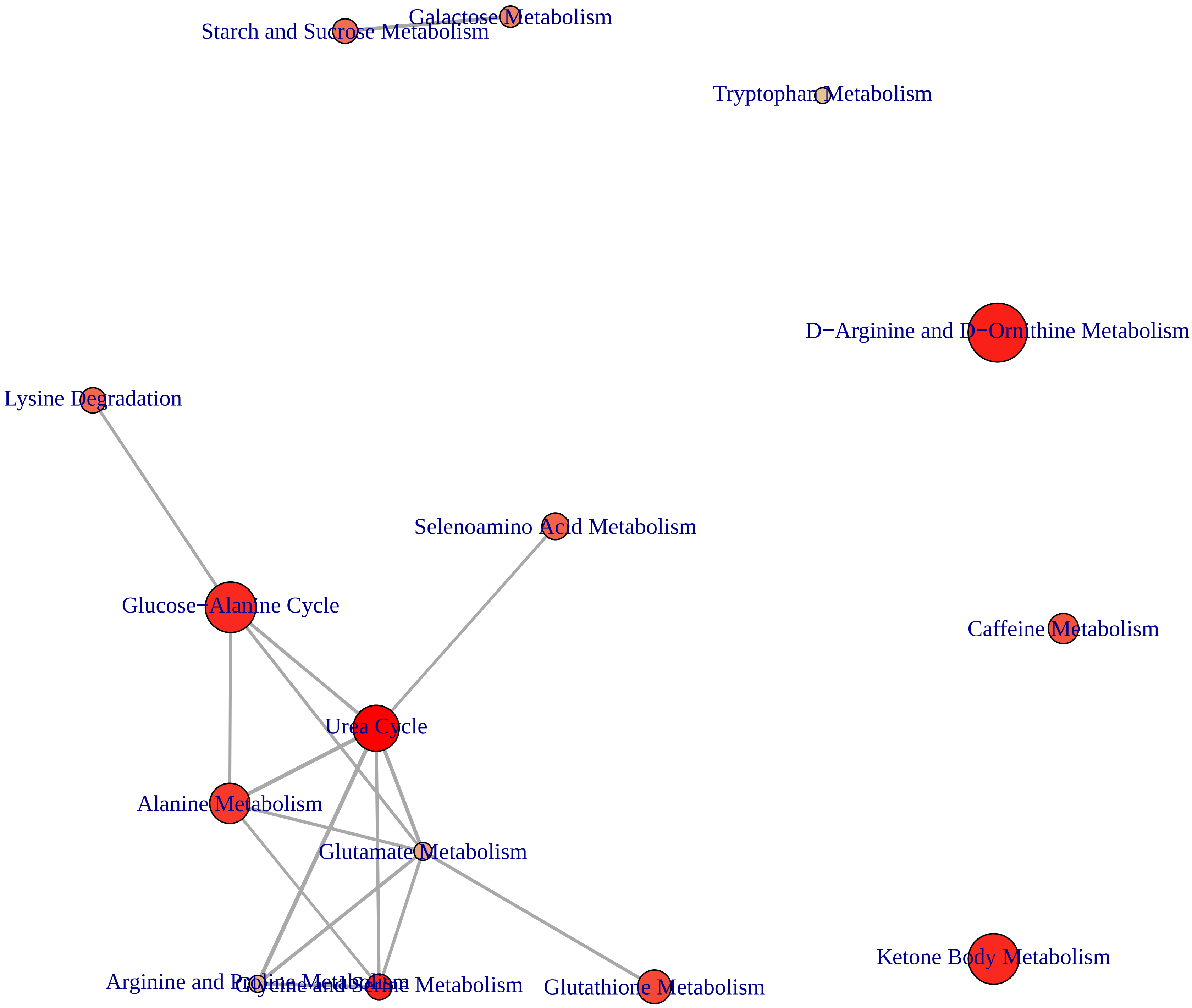
Galactose Metabolism
Starch and Sucrose Metabolism
Tryptophan Metabolism
D−Arginine and D−Ornithine Metabolism
Lysine Degradation
Selenoamino Acid Metabolism
Glucose−Alanine Cycle
Caffeine Metabolism
Urea Cycle
Alanine Metabolism
Glutamate Metabolism
Arginine and Proline Metabolism
Glycine and Serine Metabolism
Glutathione Metabolism
Ketone Body Metabolism
